# The efficacy of cannabidiol for seizures reduction in pharmacoresistant epilepsy: a systematic review and meta-analysis

**DOI:** 10.1186/s42494-024-00191-2

**Published:** 2025-03-17

**Authors:** Vinícius Gabino de Oliveira, Natália Brito de Almeida, Guilherme Corrêa Radmann, Bruno Fernandes de Oliveira Santos

**Affiliations:** 1 Medicine Departament, Tiradentes University, Aracaju, 49032-490 SE Brazil; 2 Medicine Departament, Universidade de São Paulo, São Paulo, 05508-220 SP Brazil

**Keywords:** Epilepsy, Cannabidiol, Pharmacoresistant epilepsy

## Abstract

**Background:**

Epilepsy is a neurological syndrome caused by excessive neuronal discharges, with a part of the patients being pharmacoresistant to the traditional treatment. Cannabidiol, a non-psychoactive component of *Cannabis Sativa*, shows promise as an alternative, but further research is needed to quantify its efficacy.

**Methods:**

This literature systematic review was made following the PRISMA protocol guidelines. The Google Scholar, Scielo, and PubMed/MEDLINE databases were included using the descriptors “Cannabidiol”, “Epilepsy”, and “Drug Resistant Epilepsy”. This research was registered in the Prospero platform with the identification (CRD42024479643).

**Results:**

A total of 1448 results were identified from the PubMed, Virtual Health Library, and Google Scholar databases. After applying exclusion criteria, six studies met the criteria for full-text evaluation and eligibility. The compiled analysis showed that the patients who received cannabidiol experienced a 41.0875% reduction in the total number of seizures, compared to an average reduction of 18.1% in placebo groups. This represents a 127% higher response rate for patients who received the intervention.

**Conclusions:**

Given these results, it is possible to conclude that the therapeutic response of cannabidiol is worthy of consideration in new protocols and of being added to public healthcare systems for its antiepileptic potential. However, the high efficacy rate observed in the placebo group suggests that other methods of data collection analysis may be employed.

## Background

Epilepsy is a chronic neurological syndrome characterized by at least two spontaneous epileptic seizures, which can be classified as focal or generalized depending on the affected brain regions. According to the World Health Organization (WHO), epilepsy affects an estimated 50 million people globally. Its various manifestations often cause several impairments in functional and productive capacity for individuals with the syndrome [1].

Approximately 80% of epilepsy cases are adequately treated with monotherapy [2]. However, the remaining 20% include patients classified as pharmacoresistant, who continue to experience at least one breakthrough seizure per month despite optimized treatment with two anti-seizure medications, with some cases involving thousands of seizures monthly [3]. Given the profound impact on patients’ functional quality of life and caregiver burden, multiple therapeutic options are under investigated. Among these, cannabidiol (CBD), a phytocannabinoid component of the *Cannabis Sativa* plant, has emerged as a promising candidate [4].

CBD is particularly relevant for epilepsy due to its anticonvulsant, antidepressant, antipsychotic, and neuroprotective effects, without the psychoactive effects associated with other cannabinoids [5]. Its antiepileptic potential has been studies since the 1970s [6]. In light of a significant number of well-conducted multicenter randomized studies, it becomes pertinent to collect and compile these findings to guide the evidence for CBD's efficacy in patients with pharmacoresistant epilepsy.

## Methods

### Materials and methods

This systematic review and meta-analysis were initiated to address the question: “How effective is CBD in reducing seizure frequency in patients with pharmacoresistant epilepsy?” We adopted the PICO (Patient, Intervention, Comparison, Outcome) framework to structure our inquiry. Specifically, we focused on: P: Patients diagnosed with pharmacoresistant epilepsy; I: Treatment regimens including cannabidiol; C: Pharmacoresistant patients administered a placebo as a comparison group; O: The primary outcome measured was the reduction in seizure frequency.

### Search strategy

We conducted a comprehensive search for randomized clinical trial papers published in English and Portuguese between March 2014 to January 2024 in the databases PubMed/Medline, Google Scholar, Cochrane and Scielo. The inclusion criteria were as follows: 1) use of cannabidiol as a new intervention; 2) patients diagnosed with pharmacoresistant epilepsy; 3) double-blind randomized clinical trials; and 4) availability of quantitative data to calculate changes in seizure frequency. The exclusion criteria included studies that did not meet the above categories or those for which it was not possible to obtain data on the number of convulsive seizures. The search utilized the following health descriptors: “Cannabidiol,” “Pharmacoresistant Epilepsy,” and “Epilepsy.”

We used the Mendeley Reference Manager^®^ platform for organization and removal of duplicates.

### Study selection

Two reviewers, Vinícius Gabino de Oliveira and Natália Brito de Almeida, conducted the study selection independently. Any discrepancies between their findings were resolved through consensus. Guilherme Corrêa Radmann worked as a third observer to convey their findings. The selected articles were followed the above inclusion criteria. Other works did not meet these criteria or for which it was not possible to obtain the quantity of convulsive seizures were excluded.

### Data extraction and measuring of bias risk

Using a standardized form, the two reviewers extracted the following data: study name and year, number of participants and their subgroups, study intervention strategy and results. The results consisted of the difference in the number of seizures before and after the introduction of cannabidiol or placebo interventions. Subsequently, the third observer compared the extracted results to identify and evaluate discrepancies.

### Statistical analysis

The data was compiled and tabulated in spreadsheets using Microsoft Excel on the Windows^®^ platform. The primary outcome extracted from the studies was the total number of seizures before and after the intervention. A random-effects model was used to evaluate the change in seizure frequency following the intervention. Subsequently, the meta-analysis was conducted using the RevMan^®^ 5.4 platform, generating forest plot models and calculating the *P*-value.

### Level of evidence and risk of bias

The quality of the studies was analyzed using the RevMan^®^ 5.4 criteria for assessing risk of bias, which include the following domains: Selection, Performance, Detection, Attrition, and Reporting. All selected studies were classified as having a low risk of bias due to the comprehensive description of methodologies among all of them. These data are reported in the meta-analysis graphs.

## Results

The search yielded 1448 records. After filtering based on a 10-year publication period, inclusion and exclusion criteria, and removal of duplicates, 35 articles remained for full-text analysis. A total of 6 studies were selected for analysis and comparison (Table 1). Among these, 3 focused on patients diagnosed with Dravet syndrome, while the other 3 included patients diagnosed with Lennox-Gastaut syndrome. Four of the 6 studies included subgroups for different dosages of CBD, with 3 studies comparing doses of CBD10 and CBD 20. The exception was one study that compared CBD 25 and CBD 50 for the patients diagnosed with tuberous sclerosis complex. All studies followed a similar procedure, including seizure quantity measurement, blinding process, intervention methods and periods with follow-up periods after the intervention. Some studies included branching placebo groups, while others did not. For the comparison process, placebo groups were equally divided, and their results were adjusted accordingly. Among the 6 analyzed studies, 5 reported side effects related to the CBD intervention. The most frequently reported adverse events, compared to placebo were: increased aminotransferases (risk ratio, [RR] = 11.88); sedation (RR = 4.88), and decreased appetite (RR = 3.69).

**Table 1:** Summary of included studies and reviewers’ notes

Main author	Year	Patients quantity	Intervention method	Results (reviewer 1)	Results (reviewer 2)
Devinsky [7]	2017	120 patients diagnosed with Dravet syndrome (61 received CBD20, 59 placebo)	Patients underwent CBD20 in two daily doses for 14 weeks. Both treatment and placebo groups had doses reduced by 10% per day for 10 days at the end.	The average monthly number of seizures among patients who underwent intervention was reduced by 38.9%. The placebo group showed a reduction of 13.3%. In the classification of seizure subtypes, the odds ratio favored the treatment, except for the number of absence seizures, which favored the placebo group.	The average monthly number of seizures among patients who received intervention was reduced by 38.9%. Meanwhile, the placebo group had a reduction of 13.3%.
Devinsky [8]	2018	225 patients diagnosed with Lennox-Gastaut syndrome (76 CBD20, 73 CBD10, and 76 placebo)	The treatment was administered in two daily doses, starting at 2.5 mg/kg/day and increased by 2.5 or 5 mg/kg/day depending on the assigned group until reaching the target dose.	The CBD20 group showed a reduction of 41.9% in the total number of seizures, the CBD10 group showed a reduction of 37.2%, and the placebo group had a reduction of 17.2%. The odds ratio was positive for the treatment group, with 3.85 and 3.27 for the CBD20 and CBD10 groups, respectively.	The group that received CBD20 recorded a reduction of 41.9% in total seizures, while the CBD10 group recorded a reduction of 37.2%.Furthermore, the placebo group saw a reduction of 17.2%.
Thiele [9]	2018	171 patients diagnosed with Lennox-Gastaut syndrome (86 intervention,85 placebo)	Patients were treated for 14 weeks, with 2 weeks of dose escalation to 2.5 mg/kg, followed by 12 weeks of 20 mg/kg/day in two daily doses, a 10-day titration phase with a 10% reduction in dose per day and a 4-week follow-up period.	The intervention group showed a 41.2% reduction in the total average number of seizures, while the placebo group showed a 13.7% reduction. There was also a discrepant response: 44% of participants in the intervention group reported a reduction in seizures, compared to 24% in the placebo group. Three patients in the cannabidiol group remained seizure-free during the maintenance phase.	The odds ratio for reducing the number of seizures was positive for cannabidiol, with values of 1.96, 2.76, and 3.43 for reductions of up to 25%, 50% and 75%, respectively.
Miller [10]	2020	198 patients diagnosed with Dravet syndrome (66 CBD10, 67 CBD20, 65 placebo)	The dose was administered twice daily, starting at 2.5 mg/kg/day until reaching the target dose of CBD10 or CBD20 on the 7th or 11th day, followed by 12 weeks of maintenance, 10 days of titration to dose zero, and 4 weeks of follow-up.	The CBD10 group showed a 48.7% reduction in total seizures, the CBD20 group showed a 45.7% reduction and the placebo groups showed a reduction of 29.8% and 25.7% between the two groups.	The CBD10 group was 6.56% more effective than the CBD20 with a 48.7% reduction compared to 45.7%. Furthermore, the proportion of patients who achieved at least 50% reduction in seizures was 43.9% for CBD10, 49.3% for CBD20 , and 26.2% for the sum of the placebo groups.
Privitera [11]	2021	396 patients diagnosed with Lennox-Gastaut syndrome (73 CBD10,162 CBD20 and 161placebo)	The dose was administered twice daily, starting with 2.5 mg/kg/day until reaching the target dose of CBD10 or CBD20 on the 7th or 11th day, followed by 12 weeks of maintenance, 10 days of titration to dose zero, and 4 weeks of follow-up.	The CBD10 group showed a 56.4% reduction in the total number of seizures, while the CBD20 group showed a 47.3% reduction, and the placebo group showed a 17% reduction. At the end of 28 days, 14.3% of the placebo group, 34.2% of the CBD10 group, and 41.4% of the CBD20 group achieved at least a 50% reduction in seizure frequency.	The CBD10 group recorded a 56.4% reduction in the total seizures during the intervention, while the CBD20 showed a 47.3% reduction, and the placebo group demonstrated a 20.1% reduction.
Thiele [12]	2021	224 patients diagnosed with tuberous sclerosis complex (75 CBD25, 73 CBD50, 76 placebo)	Patients started with a dose of CBD5, increasing by 5 mg/kg/day until reaching a dose of CBD25 on the 9th day and CBD50 on the 29th day, followed by 12 weeks of maintenance, 10 days of titration and 4 weeks of follow-up.	The CBD25 group showed a 47.5% reduction in total seizures, the CBD50 group showed a 26.5% reduction. The placebo groups of CBD25 and CBD50 showed reductions of 30.1% and 28%.	The group receiving CBD25 saw a 47.5% drop in total seizures, while the CBD50 group saw a 26.5% reduction. The placebo groups associated with CBD25 and CBD50 experienced a decrease of 30.1% and 28% respectively.

*CBD5* Dose of 5 mg/kg/day, *CBD10* Dose of 10 mg/kg/day, *CBD20* Dose of 20 mg/kg/day, *CBD25* Dose of 25 mg/kg/day, *CBD50* Dose of 50 mg/kg/day

Patients recorded their seizure frequency for one month under their existing treatment plan prior to the intervention. The intervention initiated with a daily dose escalation of 2.5mg to 5mg until the assigned dosage was reached, while patients continued their previous treatment plan. They then spent 12–14 weeks in the their designated branch of study, maintaining regular contact with researchers to report seizure type, frequency, adverse effects, and laboratory parameters. At the end of the intervention, the dosage was tapered by 10% per day until the intervention was completely discontinued.

**Fig. 1:**
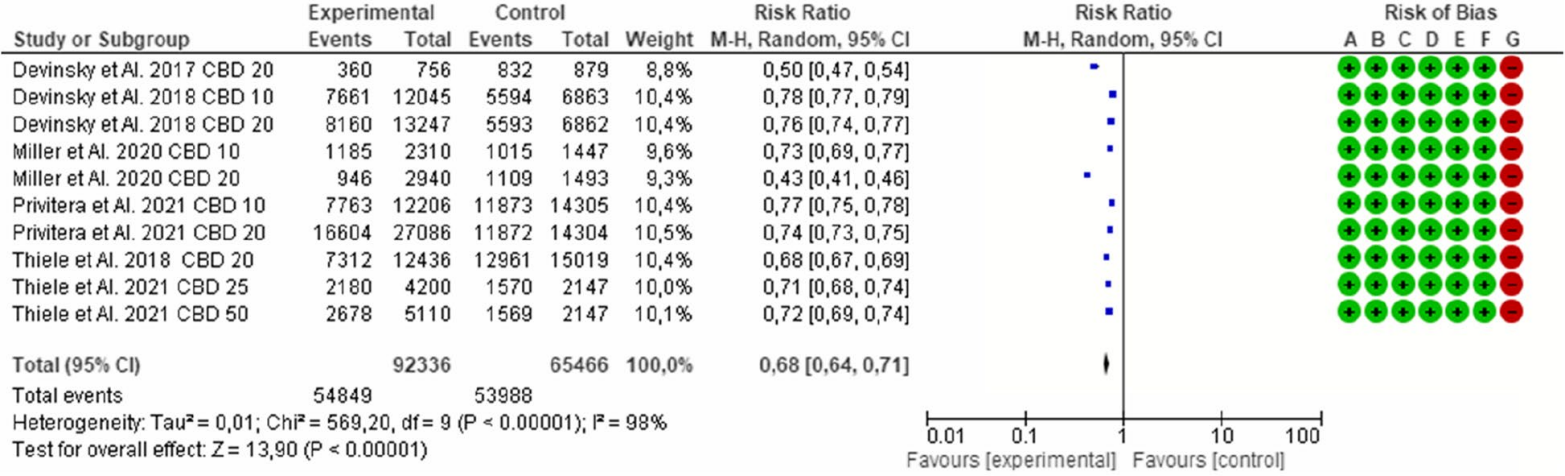
Effect of cannabidiol in all the selected studies

**Fig. 2:**
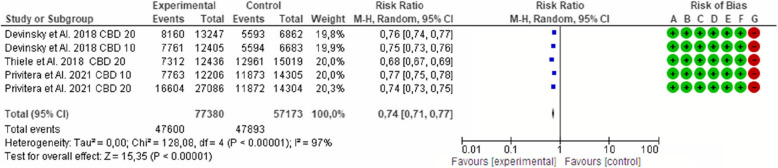
Effect of cannabidiol on the seizure frequency in Lennox-Gastaut syndrome

**Fig. 3:**
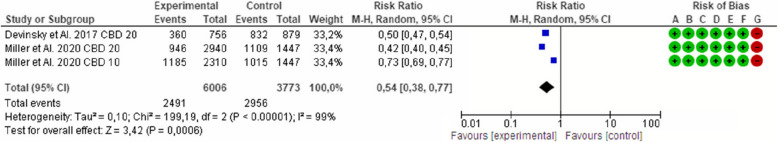
Effect of cannabidiol on the seizure frequency in Dravet syndrome

**Fig. 4:**
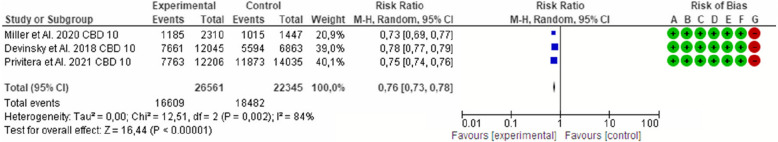
Effect of cannabidiol on the seizure frequency at a dose of 10 mg/kg/day

**Fig. 5:**
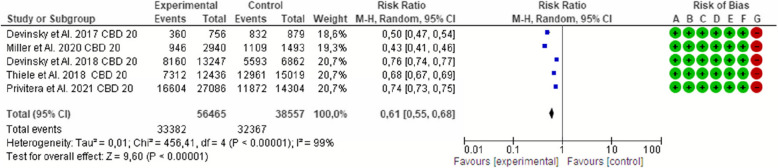
Effect of cannabidiol on the seizure frequency at a dose of 20 mg/kg/day

The main finding from the analyzed results is the efficacy of CBD in reducing the mean seizure frequency during the treatment period. Higher dose of 20 mg/kg/day showed a 12% greater improvement compared to 10 mg/kg/day across all the analyzed studies. However, 5 out of 6 studies reported that adverse effects were more prevalent at higher doses, with some participants finding the higher doses intolerable. Statistically the results were consistent across different studies, dosages and diagnosis, suggesting the efficacy of CBD in reducing seizure frequency. The higher *P-*value were observed in Figs. 1, 2, 3, 4 and 5 of the meta-analysis, which included a smaller number of studies but still demonstrated sufficiently homogenous results to be considered reliable.

## Discussion

 This systematic review initially did not account for adverse effects, nor were they part of selection criteria. However, by comparing findings with other meta-analyses and data provided on the selected articles, the three main adverse effects related to CBD treatment, expressed as percentages, were: somnolence: 24.5−28.2% (compared to 8.4–9.8% in placebo groups); decreased appetite: 20.1−25.7% (compared to 4.8–6.1% in placebo groups); diarrhea: 18.2−21.9% (compared to 8.6–9.9% in placebo groups). The safety of CBD usage primarily depends on the liver metabolizing CBD in its oil vehicle. Most analyses reported that a 3-fold increase in serum transaminases levels can be expected, and regular monitoring is recommended during CBD treatment. The suggested timeframe for monitoring liver enzymes in the analyzed studies was 3 to 6 months. Five out of the six studies reported at least one case where the intervention was suspended due to transaminase levels exceeding three times the baseline threshold. This suggests that patients with pre-existing liver injury may not be suitable candidates for CBD treatment. However, further research is needed to establish specific criteria for liver-related contraindications.

The only diagnoses repeated across different studies for comparisons were Lennox-Gastaut and Dravet syndromes, in which doses of 10 and 20/mg/kg/day were compared. For Lennox-Gastaut syndrome, the study by Devinsky et al. [8] showed better seizure control with the higher dose, while Privitera et al. [11] reported the opposite. In Dravet syndrome, only one study compared different doses, and it favored the lower dose.

Another point addressed in several studies is the variability in patient response to treatment, even among those with the same diagnosis. In Devinsky et al. [7], it was reported that some patients in the intervention group had a reduction of more than 75% in seizure frequency, and with 10 patients becoming completely seizure-free during the study period. However, 8 patients showed no improvement, and 1 patient even had an increase in the total number of seizures. The terms "Good Responders" and "Bad Responders" were introduced in this study to classify patients based on their response to treatment. "Good Responders" were defined as those with a reduction of more than 50% in the total number of seizures, while "Bad Responders" were those with a reduction of less than 50%. The terms were subsequently adopted in later studies. Although the reasons for differential patient response remain unclear, it is possible that the majority of patients qualify as "Good Responders".

Among the cases analyzed, more studies are needed to evaluate the disparity in treatment effectiveness among patients with the same diagnosis. Additionally, research should focus on identifying predictive factors to determine which patients are likely to be “Good Responders”, in order to introduce the most appropriate medication optimizing treatment outcomes.

Regarding potential biases, it is necessary to address the following topics: methodology, authorship, and response levels. 1) Methodology: the methodologies of all selected studies were highly similar. All the studies carried out a period of 28- to 30-day baseline metrics of the total number of seizures, as reported by the patients themselves or their guardians. This raises the first point of discussion: the observed reduction in seizure frequency in placebo groups, which averaged 18.1%, may be influenced by the method of seizures measurement. Because these were double-blind studies and epilepsy is a debilitating condition that causes great distress to the patient and their families, everyone has a strong desire for improvement [9]. It is even noted that many patients relocated to Ohio, USA, to participate in the study. This level of effort reflects the families' desire for a better quality of life and may partially explain the notable efficacy observed in the placebo groups. 2) Authorship: although the studies were conducted by different authors, many co-authors participated in multiple publications due to the multicenter nature of the research. For example, Elizabeth Thiele is the main author of 2 out of the 6 selected works and a co-author of 2 others. This overlap is understandable given the limited number of centers with the infrastructure and patient population necessary to conduct high-impact studies. It is important to note that there are no concerns regarding the reported methodologies, quality of the studies, or data integrity for any of the authors involved. These points are simply worth mentioning for transparency.

In Brazil, the use of CBD remains highly individualized and is not yet part of official treatment algorithms. Assessed to CBD is limited and assessed on a case-by-case basis. The factors mentioned above make it challenging to integrate CBD into standardized treatment protocols. Therapeutic testing is widely used to assess patient response and continuity of treatment, particularly due to the high cost of continuous use and limited availability within the Sistema Único de Saúde (SUS). As of 2024, CBD is not listed in the RENAME (National List of Essential Medicines), and access through SUS is only possible via court decisions or participating in special medication access programs. Furthermore, at the time of publication of this study, all the CBD used in the selected studies and those available in Brazil need to be imported, which further complicates widespread adoption due to cost and availability issues. These barriers highlight the need for discussions on improving accessibility and conducting local studies to adapt CBD use to the Brazilian context. The strengths of this study lies in its focus on an objective outcome—the reduction in seizure frequency—which allows for the compilation of data from studies of different CBD dosages. This approach supports the potential inclusion of CBD in treatment algorithm and protocols. However, the main limitation of this study is the relatively small number of studies analyzed, which, while reasonable, underscores the need for more extensive data. Additionally, all the analyzed studies were conducted over a 12-week period, leaving a gap in understanding the long-term safety and efficacy of CBD treatment.

## Conclusions

Based on the results from the analyzed studies, it can be concluded that the addition of CBD to the treatment regimen for patients with pharmacoresistant epilepsy is beneficial in most cases. The doses of 10 mg/kg/day and 20 mg/kg/day were compared in 5 out of 6 studies, with a higher dose demonstrating superior seizure control. However, the lower dose also showed significant efficacy, making it a viable option for inclusion in treatment and guidelines as well.

## Data Availability

All data used in this manuscript is available in on the analyzed and already published studies.

## Abbreviations

CBD: Cannabidiol
WHO: World Health Organization
CBD5: Dose of 5mg/kg/day of Cannabidiol
CBD 10: Dose of 10mg/kg/day of Cannabidiol
CBD 20: Dose of 20mg/kg/day of Cannabidiol
CBD25: Dose of 25mg/kg/day of Cannabidiol
CBD 50: Dose of 50mg/kg/day of Cannabidiol
RENAME: Relação Nacional de Medicamentos Essênciais (National List of Essential Medicines)
